# Psychometric properties of Greek versions of the Modified Corah Dental Anxiety Scale (MDAS) and the Dental Fear Survey (DFS)

**DOI:** 10.1186/1472-6831-8-29

**Published:** 2008-09-30

**Authors:** Trilby Coolidge, Konstantinos Nikolaos Arapostathis, Dimitris Emmanouil, Nikolaos Dabarakis, Antonis Patrikiou, Nikolaos Economides, Nikolaos Kotsanos

**Affiliations:** 1 Dental Public Health Sciences, University of Washington, Seattle, USA; 2 Department of Paediatric Dentistry, Aristotle University of Thessaloniki, Thessaloniki, Greece; 3 Department of Paediatric Dentistry, University of Athens, Athens, Greece; 4 Department of Dento-alveolar/Implant Surgery and Radiology, Aristotle University of Thessaloniki, Thessaloniki, Greece; 5 Department of Preventive Dentistry, Periodontology and Oral Implant Biology, Aristotle University of Thessaloniki, Thessaloniki, Greece; 6 Department of Endodontology, Aristotle University of Thessaloniki, Thessaloniki, Greece

## Abstract

**Background:**

A growing body of literature describes the performance of dental fear questionnaires in various countries. We describe the psychometric properties of Greek versions of the Modified Dental Anxiety Scale (MDAS) and the Dental Fear Survey (DFS) in adult Greek patients.

**Methods:**

Greek versions of the MDAS and DFS were administered to two samples of adult dental patients. In the first sample, 195 patients attending one of three private practice dental offices in a large city in Greece completed the questionnaires in the waiting room before dental treatment. After treatment, their dentists (who did not know how the patients had answered the questionnaire) rated their anxiety during dental treatment. In the second sample, 41 patients attending a Greek university dental school clinic completed the questionnaire twice at two separate visits, in order to provide test-retest data. Cronbach's alpha was used to compute the internal consistencies, while Spearman's rho was used to compute the test-retest reliabilities. Construct validity was assessed by correlating the responses to the MDAS and DFS by Spearman's rho. Spearman's rho was also used to examine the criterion validities, by comparing the questionnaire responses with the dentists' ratings of anxiety.

**Results:**

The internal consistencies for the MDAS were 0.90 and 0.92 in the two samples; for the DFS, the internal consistencies were 0.96 in both samples. The test-retest reliabilities were 0.94 for the MDAS and 0.95 for the DFS. The correlation between the two questionnaires was 0.89. The patients' responses to both questionnaires were significantly related to the dentists' ratings of their anxiety during dental treatment (both p values <0.001).

**Conclusion:**

The results indicate that the Greek versions of the MDAS and DFS have good internal consistencies and test-retest reliabilities, as well as good construct and criterion validities. The psychometric properties of the Greek versions of these questionnaires appear to be similar to those previously reported in other countries.

## Background

Dental fear affects a considerable number of patients and is linked to avoidance of dental treatment, often resulting in pain and the need to undergo more invasive treatment when patients do come to the dentist [1]. Further, the tendency to receive symptom-oriented treatment (rather than preventive treatment) is related to higher levels of dental fear, providing evidence for a "vicious cycle" in which dental fear continues to be predictive of avoidance, greater dental treatment needs, symptom-oriented care, and continued fear [2]. When fearful patients do appear for dental treatment, they may pose special treatment considerations for the practitioner [1].

Clinicians and researchers alike have the need for valid measures of dental fear. Questionnaires have several advantages over other methods of assessing fear. First, they are quick and inexpensive to administer and score. Second, they have high face validity, making them appropriate tasks for patients and research subjects to engage in. Because of these qualities, several measures of dental fear have been developed for adult and child patients [3,4].

Perhaps the two most frequently used adult questionnaire measures of dental fear and anxiety are the Dental Anxiety Scale [DAS; [5]] and the Dental Fear Survey [DFS; [6]]. Both were originally developed in English. The original DAS is a 4-item questionnaire, asking individuals to rate their anxiety as they imagine approaching four dental situations, such as sitting in the waiting room anticipating dental treatment. The Modified Dental Anxiety Scale [MDAS, [7]] was developed to improve the psychometrics and content validity of the original DAS by adding an item about receiving dental injections, and ordering the potential answers to each item so that they range from least to greatest level of anxiety [3,4]. The MDAS has been found to be reliable and valid in several samples from England, Scotland, Wales, Ireland, Finland, Dubai, Brazil, and Turkey, as well as in a sample of Spanish-speaking individuals in the United States [7-12].

The original DFS contained 27 items [13], which the authors later reduced to 20 [3]. The items assess a broader array of dental stimuli than the MDAS, such as seeing the drill, smelling the dental office, and the like. In addition, the respondent is asked to rate specific physiological responses to dental stimuli, such as muscle tension and increased breathing rates. Two items assess avoidance of dental appointments due to fear, and one item asks for an overall rating of fear of dental work. The DFS has been found to be reliable and valid in samples of college students and dental patients [3]. The measure has been translated into a number of languages, including Danish, Swedish, Norwegian, Hungarian, Brazilian, Turkish, Spanish (for Hispanics in the United States), Castilian (Spanish spoken in Spain), Chinese and Malay versions [12,14-21].

The criterion validity of the DFS has often been assessed by comparing groups of dental phobics with non-phobics, finding that phobics score higher than non-phobics [e.g., [16]]. Similarly, MDAS scores have been found to be higher in groups of dental phobics, compared with other individuals [e.g., [7]]. In situations where patients' classification as dentally phobic has not already been determined, alternate methods of validation have been used. For example, Corah compared patients' scores on the original DAS with independent dentists' ratings of the patients' anxiety during treatment [5].

Psychiatrists have noted that there may be cultural differences in the manifestation of various anxiety disorders [22]. This also appears to be the case with dental fear. For example, Humphris and colleagues [8] found cultural differences in the proportions of patients with high dental anxiety, as well as which dental stimuli were rated as the most feared. This indicates that it would be useful to study dental fear within each culture of interest, rather than extrapolate findings from other cultures.

Greek dentists have noticed fearful behavior in some of their patients but have not had a Greek version of a standard fear questionnaire to accurately assess fear. In addition, researchers have wished to study the effectiveness of fear-reduction techniques in Greek patients, but have likewise been unable to quantify change due to the lack of valid Greek fear measures. Thus, developing and testing Greek dental fear questionnaires would assist dentists and researchers who work with this population. In this paper, we report on the development and psychometric properties of the Greek versions of two dental fear measures for adults, the MDAS [7], and the DFS [6].

## Methods

The study was approved by Institutional Review Boards at Aristotle University of Thessaloniki, Thessaloniki, Greece and the University of Washington, Seattle, Washington, USA.

Two groups of adult dental patients in a large city in Greece were selected for this study. The first group consisted of consecutive patients seen by three general dentists in private practice over a six month period, and was used to assess the internal consistencies, construct validity and criterion validity of the measures. Because few (< 10) of these patients returned to the dentist for a second appointment during the six month period, we added a second group of patients. The second group consisted of a subset of patients seen in a dental school clinic, and was used to assess the test-retest reliabilities of the measures. Potential participants were identified if the researchers determined that they would need two dental appointments approximately 1 to 3 weeks apart. In order to sample approximately 100 patients for the test-retest analysis, recruitment took place over a 13-month period (including 10 months total when the clinic was open, and 3 months when the clinic was closed for the summer). Because of the large number of student dentists, it was not practical to calibrate them on the criterion validation methods, and thus the criterion validity of the questionnaires was not assessed in this group of participants.

There were two questionnaires used in this study, one for dental patients and another for dentists. The patients' questionnaire consisted of the MDAS, the DFS, and demographic questions. The MDAS and DFS items are answered on 5-point scales, which are then summed to create an overall score. The total possible scores on the MDAS range from 5 to 20, while the total possible scores on the DFS range from 20 to 100. On both measures, higher scores refer to higher levels of dental fear. All items were translated into Greek by a bilingual dentist, then independently back-translated by a second bilingual dentist. Minor corrections were made until the two dentists agreed on the final wording. (The Greek translations of the MDAS and the DFS are available from the first author.)

The dentists' questionnaire included one item for rating the patient's anxiety level during the treatment, on a 5-point scale ranging from "Not at all anxious" to "Extremely anxious". The three private practice dentists scored pilot patients on the anxiety rating scale until they developed sufficient agreement (kappas ranged from 0.81 to 0.84) with one of the senior dentist-researchers in the study. Following this, recruitment began in the private practices.

Potential participants were approached in the waiting room. Participants gave written consent. Participants filled out the questionnaire in the waiting room, before their dental appointment. Following the dental appointment, the dentist (who was unaware of the patient's answers to the questionnaire) rated the patient's level of anxiety during the dental procedure. Participants were not paid for their participation.

In the University setting, four clinics were identified whose patients frequently required two appointments. These clinics typically offered periodontal treatment, root canal treatment, restorations, and extractions. Patients are seen by student dentists under faculty supervision. To be eligible, potential participants were chosen from among patients judged most likely to return to the same clinic within a three-week period and be seen by the same student dentist under supervision by the same faculty member. These criteria were chosen to maximize the probability that the patient would receive the second questionnaire at the second dental appointment. Potential participants were identified by one of the senior dentist-researchers in the study. Potential patients were then approached in the waiting room. After giving written consent, they completed the questionnaire in the waiting room. Patients who returned for a second appointment during the time frame of the study completed the questionnaire a second time. Participants were not paid to participate.

Analyses were carried out with SPSS version 14.0 for Windows (SPSS Inc., Chicago, IL, USA). Participants who completed both the MDAS and the DFS were retained for the analyses. In addition to descriptive statistics, Cronbach's alpha was used to calculate internal consistencies, and t-tests were used to compare males' and females' scores on the measures. Because the distributions of the fear measures were skewed (most participants had low levels of fear), Spearman's rho was used to compare scores on the two measures to examine construct validity, as well as to calculate the test-retest statistic. Spearman's rho was also used to compare the scores on the fear measures and the dentists' ratings of patient anxiety during treatment, to examine criterion validity. Because fewer university clinic patients returned for a second appointment than anticipated, we also compared those who did and those who did not return on dental fear scores at the first appointment (t-test), age (t-test), and gender (Chi square), to look for possible group differences.

## Results

In the three private practice offices, approximately 205 consecutively-seen adult patients were approached to participate in the study, and 195 (95%) patients agreed. Of these, 148 completed both the MDAS and DFS. Their mean age was 40.3 years (SD = 12.5, median = 38.0, range = 18–76), and 51.4% were male. MDAS and DFS scores for all participants, and for males and females separately, are shown in Table 1. The mean (SD) scores for the MDAS were 10.91 (4.79) for all participants, 9.80 (4.20) for the males, and 12.18 (5.11) for the females, with the difference between males and females being statistically significant (t = 3.070, df = 135.72, p = 0.003). The overall mean (SD) for the DFS was 39.32 (17.07). The mean (SD) scores for males and females were 36.41 (15.03) and 42.85 (18.54), respectively; the difference between males and females was statistically significant (t = 2.30, df = 134.82, p = 0.023).

**Table 1:** Modified Dental Anxiety Scale (MDAS) Scores, Dental Fear Survey (DFS) Scores, and Dentists' Ratings of Anxiety During Dental Treatment in Private Practice Patients

	Mean	Median	SD	Range
MDAS: All Participants	10.91	10.00	4.79	5–25
MDAS: Males	9.80	9.00	4.20	5–22
MDAS: Females	12.18	11.00	5.11	5–25
DFS: All Participants	39.32	34.00	17.07	20–92
DFS: Males	36.41	31.00	15.03	20–83
DFS: Females	42.85	38.00	18.54	20–92
Dentists' Ratings: All Participants	1.90	2.00	0.95	1–5
Dentists' Ratings: Males	1.77	2.00	0.82	1–4
Dentists' Ratings: Females	2.06	2.00	1.07	1–5

MDAS possible range = 5 (no fear) – 25 (highest level of fear)

DFS possible range = 20 (no fear) – 100 (highest level of fear)

Dentists' Ratings possible range = 1 (Not at all anxious) – 5 (Extremely anxious)

The internal consistencies (Cronbach's alpha) were 0.90 and 0.96 for the MDAS and DFS, respectively. The correlation (Spearman's rho) between the two questionnaires was 0.89 (p < 0.001).

The dentists' ratings of anxiety during dental treatment are also shown in Table 1. There was a trend for females to be rated as more anxious (t = 1.804, df = 131.335, p = 0.073).

The correlations between the dentists' ratings and the patients' scores on the fear questionnaires are shown in Table 2. These correlations ranged from 0.49 to 0.66, and were significant at p < 0.001.

**Table 2:** Correlations (Spearman's rho) Between Dentists' Ratings of Anxiety and Cooperation and Scores on Modified Dental Anxiety Scale (MDAS) and Dental Fear Survey (DFS)

	MDAS	DFS
Anxiety: All Participants	0.58***	0.61***
Anxiety: Males	0.49***	0.53***
Anxiety: Females	0.64***	0.66***

* = p < 0.05

** = p < 0.01

*** = p < 0.001

In the dental school clinics, 98 patients were judged to be eligible for this sample and were approached in the waiting room. Of them, 95 agreed to participate, and 82 completed the MDAS and DFS at the first visit. Their average age was 34.3 (SD = 13.1, median = 31.0, range = 19–96), and 48.1% were male. As shown in Table 3, the mean MDAS score was 10.48 (SD = 5.11, median = 8.00, range = 5–25). The mean DFS score was 39.76 (SD = 17.49, median = 34.50, range = 20 – 96). The internal consistencies (Cronbach's alpha) for the two scales were 0.92 (MDAS) and 0.96 (DFS).

**Table 3:** Modified Dental Anxiety Scale (MDAS) and Dental Fear Survey (DFS) Scores in University Dental Clinic Patients at First Appointment

	Mean	Median	SD	Range
MDAS: All Participants	10.48	8.00	5.11	5–25
MDAS: Males	8.95	8.00	4.50	5–25
MDAS: Females	11.83	10.50	5.30	5–25
DFS: All Participants	39.76	34.50	17.49	20–96
DFS: Males	36.34	33.00	15.45	20–86
DFS: Females	43.05	37.50	18.61	20–96

MDAS possible range = 5 (no fear) – 25 (highest level of fear)

DFS possible range = 20 (no fear) – 100 (highest level of fear)

Half (41) of the patients seen in the dental school clinics returned for a second dental visit within the time frame of the study. The interval between appointments ranged from 7 to 68 days; 14 days was the modal interval, and 79.5% of the second appointments were held within 3 weeks of the first appointment. There were no statistically significant differences in age, gender, or level of dental fear (scores on MDAS or DFS at the first appointment) between those who returned for a second appointment and those who did not.

At the second appointment, all 41 patients completed the MDAS a second time. The mean MDAS score for the second administration was 10.32 (SD = 5.17, median = 7.00, range = 5–23). Thirty nine of the 41 also completed the DFS at the second appointment. The mean DFS score for the second administration was 37.09 (SD = 17.04, median = 31.00, range = 20–75). The test-retest statistics (Spearman's rho) for the two scales were 0.94 for the MDAS (p < 0.001, n = 41) and 0.95 for the DFS (p < 0.001, n = 39).

## Discussion

In this study, we found good evidence for the internal consistencies and test-retest reliabilities of the Greek translations of both the MDAS and DFS. In addition, we found good evidence for the construct and criterion validities of the Greek versions of both measures. Thus, it appears that the Greek versions of these measures operate in similar ways as they have in other languages.

A dentist whose patient population includes Greeks may wish to use one or both measures as a method of assessing dental fear. As other authors have commented, the MDAS has the advantage of being brief, and therefore may be preferred for clinic purposes [3,4]. On the other hand, the DFS assesses more stimuli, and its increased comprehensiveness may be preferred for research purposes [3,4]. In addition, fearful patients may appreciate the DFS as it is more likely to include items which describe specific stimuli they find to be anxiety-provoking.

We found that Greek women scored significantly higher on both fear measures, compared with men, which has been frequently noted in other studies of dental fear [1]. Vassiliou and colleagues found that Greek women are more anxious in general, compared with Greek men [23], which is consistent with much other research on gender differences in anxiety [24]. Our results indicate that dentists working with Greek populations can expect that females will be more likely to have higher levels of dental fear, compared with males.

The validity of the MDAS and DFS has often been assessed by comparing groups of dentally fearful and non-fearful patients. To our knowledge, there is no dental clinic in Greece specializing in treating dentally fearful patients. Therefore, we did not have access to a sample of previously-identified fearful patients to use in a group comparison. Instead, we used dentists' ratings of the patients' anxiety during treatment to assess criterion validity. Dentists' ratings have been used in the original validation of the DAS [5]. This method has also been used in the validation of other measures of dental fear. For example, childrens' scores on the Children's Fear Survey Schedule Dental Subscale (CFSS-DS) have been found to be related to dentists' ratings of fearful and cooperative behavior during dental treatment [25-28]. Our results indicate that patients' scores on the two measures are significantly related to dentists' independent ratings during treatment. Nevertheless, a comparison of Greek patients previously-identified as fearful with patients who are not fearful would add to the criterion validity information about these measures; furthermore, such a study could explore the sensitivity and specificity of the measures.

Although we found evidence for very high test-retest reliability in this study, only half of the patients returned for a second appointment during the period of data collection. It is unclear why this was so. It is possible that some of the patients completed their treatment needs in a single appointment. Comparisons of those who did and did not return to the same student dentist for the second appointment found no differences in age, gender, or levels of dental fear between the two groups, suggesting that the smaller sample who completed the second questionnaire was similar to those patients who did not. Nevertheless, our attrition rate of 50% raises the possibility that those who did not return for a second appointment with the same dentist may have been different in some other important way, compared with those who did. It would be useful to study the test-retest reliability in additional samples of patients in which the attrition rate is lower.

## Conclusion

We found that the Greek versions of the MDAS and DFS have good psychometric properties. This means that dentists and researchers working with Greek-speaking populations may use either or both measures to assess levels of dental fear. The trend for dentists to rate Greek females as being more anxious during dental treatment, compared with males, may be consistent with our finding that Greek females have significantly higher levels of dental fear. In the future, it would be useful to reassess the test-retest reliabilities in a sample with less attrition. It would also be useful to compare the questionnaire results of patients who have previously been identified as fearful and those who are not fearful, to further assess the Greek versions.

## Competing interests

The authors declare that they have no competing interests.

## Authors' contributions

TC designed the study, performed the data analyses, and wrote the draft and revisions of the manuscript. KNA and DE translated the questionnaires into Greek. KNA also calibrated the dentists, and oversaw data collection and procedures at the private practices. ND, AP, and NE collected data at the private practices. NK oversaw data collection and procedures at the university dental clinic. All authors approved the manuscript.

## Pre-publication history

The pre-publication history for this paper can be accessed here:


